# Spatial epidemiology and spatial ecology study of worldwide drug-resistant tuberculosis

**DOI:** 10.1186/1476-072X-10-50

**Published:** 2011-08-03

**Authors:** Yunxia Liu, Shiwen Jiang, Yanxun Liu, Rui Wang, Xiao Li, Zhongshang Yuan, Lixia Wang, Fuzhong Xue

**Affiliations:** 1 Department of Epidemiology and Health Statistics, School of Public Health, Shandong University, Jinan 250012, China; 2 Tuberculosis Prevention and Control Center, Chinese Center for Disease Control and Prevention, Beijing 102206, China

**Keywords:** drug-resistant tuberculosis, epidemiology, risk factors, Kriging method, partial least square path modeling (PLS-PM), geographical weighted regression (GWR)

## Abstract

**Background:**

Drug-resistant tuberculosis (DR-TB) is a major public health problem caused by various factors. It is essential to systematically investigate the epidemiological and, in particular, the ecological factors of DR-TB for its prevention and control. Studies of the ecological factors can provide information on etiology, and assist in the effective prevention and control of disease. So it is of great significance for public health to explore the ecological factors of DR-TB, which can provide guidance for formulating regional prevention and control strategies.

**Methods:**

Anti-TB drug resistance data were obtained from the World Health Organization/International Union Against Tuberculosis and Lung Disease (WHO/UNION) Global Project on Anti-Tuberculosis Drug Resistance Surveillance, and data on ecological factors were collected to explore the ecological factors for DR-TB. Partial least square path modeling (PLS-PM), in combination with ordinary least squares (OLS) regression, as well as geographically weighted regression (GWR), were used to build a global and local spatial regression model between the latent synthetic DR-TB factor ("DR-TB") and latent synthetic risk factors.

**Results:**

OLS regression and PLS-PM indicated a significant globally linear spatial association between "DR-TB" and its latent synthetic risk factors. However, the GWR model showed marked spatial variability across the study regions. The "TB Epidemic", "Health Service" and "DOTS (directly-observed treatment strategy) Effect" factors were all positively related to "DR-TB" in most regions of the world, while "Health Expenditure" and "Temperature" factors were negatively related in most areas of the world, and the "Humidity" factor had a negative influence on "DR-TB" in all regions of the world.

**Conclusions:**

In summary, the influences of the latent synthetic risk factors on DR-TB presented spatial variability. We should formulate regional DR-TB monitoring planning and prevention and control strategies, based on the spatial characteristics of the latent synthetic risk factors and spatial variability of the local relationship between DR-TB and latent synthetic risk factors.

## Background

Tuberculosis (TB) is a major cause of illness and death worldwide. The World Health Organization (WHO) estimated that there were 14 million prevalent TB cases (range, 12 million-16 million), 1.3 million deaths among HIV-negative people and 0.38 million deaths among HIV-positive people in 2009 [1]. Recently, drug-resistant TB (DR-TB), and especially multidrug-resistant TB (MDR-TB), has emerged as an increasingly important factor in TB deaths [2]. According to a WHO report, DR-TB has spread worldwide and has become a serious public health problem that threatens the success of the directly-observed treatment strategy (DOTS), a treatment approach recommended by WHO for the detection and cure of TB, as well as global TB control [3]. Among TB patients notified in 2009, an estimated 250,000 had MDR-TB [1]. DR-TB is caused by various factors, including not only factors at individual level (e.g., sex [3], genetic susceptibility [4], occupation [5], previous treatment [6-11], socioeconomic status [12-14], etc.), but also factors at ecological level (i.e., environment factors, including natural factors and social factors [15]). Therefore, it is essential to investigate in depth the risk factors for DR-TB prevention and control, especially the ecological factors.

From 1994, WHO, the International Union Against Tuberculosis and Lung Disease (The Union) and other partners have launched the Global Project on Anti-Tuberculosis Drug Resistance Surveillance (the Global Project) [16]. Since the establishment of the Global Project, 114 countries (59% of all countries) have been covered, and data on drug resistance have been systematically collected and analyzed [2]. The results indicate that Central and Western Europe have the lowest proportions of resistance to any TB drug and the lowest MDR-TB, followed by African countries and then The Americas, with moderate proportions of resistance in the Eastern Mediterranean and South-East Asia regions, followed by the Western Pacific region. Proportions of resistance to any TB drug and MDR-TB are highest globally and for all first-line drugs in Eastern Europe. Furthermore, important variations exist within different regions [2]. This suggests that ecological causes (specifically, climate and geography, TB epidemiological factors and socioeconomic factors, etc.) for DR-TB vary in different regions. Spatial examination of these risk factors for DR-TB would play an essential role in developing regional prevention measures and control strategies.

However, the variables of climate and geographical factors, TB epidemiological factors and socioeconomic factors, etc. usually show spatial autocorrelation and obvious spatial heterogeneity [17,18], which is difficult for the traditional multivariable model (e.g. global ordinary least square (OLS) regression [19]) to deal with. We therefore introduced geographical weighted regression (GWR) [20] to assess the spatial heterogeneity in the putative relationships between DR-TB and its risk factors. As the variables involved in this study presented characteristics of high-dimension, non-normality, small sample size and multicollinearity, we first proposed partial least square path modeling (PLS-PM) [21,22] to extract the latent synthetic DR-TB factors from the DR-TB vector, and latent synthetic risk factors from the ecological factors vector; then we constructed the structural equation model (SEM) to analyze the complex causal relationship between the latent synthetic DR-TB factors and latent synthetic risk factors. Furthermore, the GWR model was employed to analyze the local spatial heterogeneity in the estimated relationships between the latent synthetic DR-TB factors and latent synthetic risk factors. All the maps in this study were created by ArcGIS (v9.0) [23].

## Methods

### Setting

**Figure 1:**
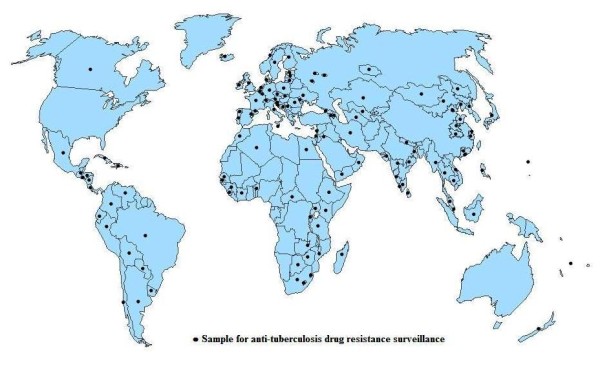
**Locations of 126 regions for anti-tuberculosis drug resistance surveillance**.

In 2008, the fourth report [2] of the WHO/UNION Global Project on Anti-Tuberculosis Drug Resistance Surveillance was published, which summarized data from 114 countries between 1994 and 2007. 109 countries (covering 126 regions, Figure 1) reported data on first-line anti-TB drug resistance among new cases, including prevalence of resistance to isoniazid (H), rifampicin (R), streptomycin (S) and ethambutol (E). We used mono-drug resistance (resistance to a single drug, H, R, E or S: Mono-rate), multidrug resistance (resistance to, at least H and R, including HR, HRE, HRS and HRES: MDR-rate), and poly-drug resistance (resistance to several drugs, excluding combined resistance to H and R, including HE, HS, RE, RS, ES, HES and RES: Poly-rate) to reflect the prevalence of drug resistance. The present study was carried out using surveillance regions as units of analysis.

### Data sources

The anti-TB drug resistance data was extracted from the fourth report, including Mono-rate, MDR-rate and Poly-rate of DR-TB in 126 regions. A worldwide spatial database on ecological factors was compiled, including climate, geography, TB epidemic, the effects of DOTS, health expenditure and health service factors, etc. The climatic and geographic data (including annual precipitation, annual atmospheric temperature, temperature climate zone, geography climatic zone and geography latitude) of the 126 regions were collected from the World Climate website, and Table 1 shows the value assignment. The other ecological factors (Table 2), including TB epidemic, the effects of DOTS, health expenditure and health service, etc. were extracted from the Health Resource Database of the WHO website, the Government websites of some countries or regions, the internet and relevant references, etc. Considering the hysteresis quality of drug resistance, the collected data on ecological factors were 3-5 years earlier than the surveillance time of drug resistance. The anti-TB drug resistance data and ecological factors data together with their value assignment are provided as a supplement (see Additional file 1).

**Table 1:** Value assignment of the climatic and geographic factors

Geographical climate index	Variable	Assignment
Annual precipitation (AP)	0 mm ≤ AP < 200 mm	1
	200 mm ≤ AP < 500 mm	2
	500 mm ≤ AP < 1000 mm	3
	1000 mm ≤ AP < 2000 mm	4
	2000 mm ≤ AP	5

Annual atmospheric temperature (AAT)	30°C ≤ AAT	6
	20°C ≤AAT < 30°C	5
	10°C ≤ AAT < 20°C	4
	0°C ≤AAT < 10°C	3
	-10°C ≤ AAT < 0°C	2
	-20°C ≤ AAT < -10°C	1

Temperature climate zone (TCZ)	frigid zone	1
	subfrigid zone	2
	temperate zone	3
	subtropical zone	4
	tropical zone	5

Geography climatic zone (GCZ)	continental climate	1
	transitional climate	2
	oceanic climate	3

Geography latitude (GL)	0° ≤ GL < 25°	3
	25° ≤ GL < 50°	2
	50° ≤ GL < 75°	1

**Table 2:** Ecological influencing factors

Ecological influencing factors index	
x1	TB case notification rates (per 100 000 population)
x2	Prevalence of TB (per 100 000 population)
x3	TB mortality among HIV-negative people (per 100 000 population)
x4	Population with sustainable access to improved rural sanitation (percent)
x5	1-year-olds immunized with three doses of DTP3 (%)
x6	1-year-olds immunized with MCV (%)
x7	Life expectancy at birth (years)
X8	Total expenditure on health as percentage of gross domestic product
X9	Per capita total expenditure on health at average exchange rate (US$)
x10	Per capita government expenditure on health at average exchange rate (US$)
x11	New smear-positive TB treatment success under DOTS (%)
x12	TB treatment success under DOTS (%)

### Analysis of the complex relationship between DR-TB and ecological factors

To explore the latent structure of the DR-TB vector and the ecological risk factors vector, exploratory factor analysis (EFA) [19] was used to extract the latent synthetic DR-TB factors and latent synthetic risk factors by SAS9.0 software. Based on the results of EFA, SEM was constructed to show the complex relationship between the latent synthetic DR-TB factors and latent synthetic risk factors. Because of the non-normal distribution, small sample size and multicollinearity of the data, the PLS algorithm was chosen to set up SEM, named as PLS-PM [21]. It is a component-based estimation method, which is an iterative algorithm that separately analyzes the blocks of the measurement model and then estimates the path coefficients in the structural model. PLS-PM is regarded as a "soft modeling" approach, without strong assumptions for the distributions, the sample size and the measurement scale, and has been applied extensively, especially in customer satisfaction studies [22]. Based on the software review by Temme et al. (2006) [24], the software SmartPLS version 2.0M3 [25] was chosen to conduct the analysis. SmartPLS supports graphical modeling and carries out the bootstrapping procedure to generate significance measures. In this research, the path weighting scheme was implemented for the inner estimate of the standardized latent variable in PLS analysis, and the resampling number was specified as 1000 in bootstrapping. Furthermore, the latent synthetic DR-TB factors and latent synthetic risk factors scores were estimated for further analysis.

### Detection of spatial dependence relationship between DR-TB and ecological factors

To explore the spatial dependence relationship between DR-TB and its ecological risk factors, PLS-PM, in combination with OLS regression as well as GWR, was used to build the global and local spatial regression model between the latent synthetic DR-TB factors and latent synthetic risk factors. PLS-PM was firstly used to estimate the latent synthetic DR-TB factors and latent synthetic risk factors for each region as above. Then, the ordinary Kriging interpolation [26] was used to obtain the predicated values of the latent synthetic DR-TB factors and latent synthetic risk factors. Finally, by using Spatial Analysis software in Macroecology (SAM v4.0) [27], OLS regression [19] and GWR [20,28,29] were used to set up the global and local spatial regression models between the latent synthetic DR-TB factors and the latent synthetic risk factors, respectively.

As a virtually unbiased method in an interpolation situation, the Kriging model has several advantages over other interpolation and smoothing methods, and has been used to create maps of geographic disease clines in many studies [30,31]. In this study, after the Kriging maps were created by ArcGIS, the Natural Breaks (Jenks) method [32] was used to classify the latent synthetic DR-TB and risk factor clines. Unlike conventional OLS regression, which may only produces a single regression equation to summarize global relationships between DR-TB and ecological synthesis factors, the GWR, whose regression coefficients are allowed to vary spatially, can generate spatial dependence that express the local spatial variation between them dynamically. GWR has been successfully applied in spatial epidemiology [27,33-36] and in spatial ecology [26,37,38].

## Results

### Latent synthetic risk factors and DR-TB factors

From five climatic and geographic factors (Table 1), two latent synthetic risk factors were extracted, which could explain about 87.17% of the total variance for these factors. The first, named as "Temperature", was reflected by annual atmospheric temperature (ATT), temperature climate zone (TCZ) and geography latitude (GL). The second, named as "Humidity", was described by annual precipitation (AP) and geography climatic zone (GCZ). Based on the value assignment of the climatic and geographic factors (Table 1), the larger the "Temperature", the hotter the climate; and the larger the "Humidity", the wetter the climate.

Four latent synthetic factors were extracted from the TB epidemic situation, the effects of DOTS, health expenditures, etc., which could explain 86.09% of the total variance for the twelve ecological risk factors (Table 2). The first, named as "TB Epidemic", was reflected by TB case notification rates (x1), prevalence of tuberculosis (x2), and TB mortality among HIV-negative people (x3). The second, named as "Health Service", consisted of population with sustainable access to improved rural sanitation (x4), 1-year-olds immunized with diphtheria-tetanus-pertussis (DTP3) (x5), 1-year-olds immunized with meningococcal conjugate vaccine (MCV) (x6) and life expectancy at birth (x7). The third, named as "Health Expenditure", was composed of total expenditure on health as percentage of gross domestic product (x8), per capita total expenditure on health at average exchange rate (x9) and per capita government expenditure on health at average exchange rate (x10). The fourth, named as "DOTS effect", was described by new smear-positive TB treatment success under DOTS (x11) and TB treatment success under DOTS (x12). Obviously, the larger the "TB Epidemic", the more serious the TB epidemic situation; the larger the "Health Service", the higher quality the health service; the larger the "Health Expenditure", the greater the health investment; the larger the "DOTS Effect", the better the effect of DOTS.

From Mono-rate, MDR-rate and Poly-rate, the latent synthetic factor named as "DR-TB" was extracted to reflect the prevalence of drug resistance, which could explain 70.09% of the total variance. It can be seen that the larger the "DR-TB", the more serious the epidemic situation.

### Complex relationship between "DR-TB" and ecological factors

**Figure 2:**
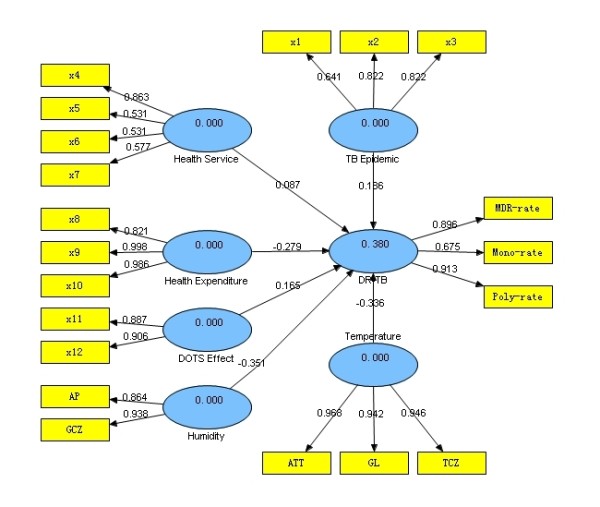
**PLS path model of DR-TB rates with ecological factors**.

Figure 2 shows the PLS path model of DR-TB rates with ecological factors, in which reflective mode was used to relate the manifest variables (ecological factors) to their latent variables (latent synthetic risk factors). It can be seen that the six latent synthetic risk factors could explain 38% of the total variation of the "DR-TB" factor (see Figure 2). Among them, the "Humidity" factor had the largest effect, with a standardized path coefficient -0.351, i.e., there was a negative relationship between "Humidity" and "DR-TB", and the larger the "Humidity", the lower the prevalence of DR-TB. Both "Temperature" and "Health Expenditure" factors had a negative effect on "DR-TB", with standardized path coefficients -0.336 and -0.279, respectively. "TB Epidemic", "DOTS Effect" and "Health Service" factors all had a positive influence on "DR-TB", with standardized path coefficients 0.186, 0.165 and 0.087, respectively. Table 3 and Table 4 show the bootstrapping test results for outer loading of the measurement model and path coefficient of the structure model, which demonstrated that in the measurement model, most loadings of the manifest variables except x5 and x6 were significant at 0.20 level (*P *< 0.20); in the structure model, all path coefficients except "Health Service" were significant at 0.20 level (*P *< 0.20). Therefore, the analysis indicated that the latent synthetic risk factors "TB Epidemic", "Health Expenditure", "DOTS effect", "Humidity" and "Temperature" had major effects and played an important role in drug resistance, while "Health Service" had a little effect.

**Table 3:** Bootstrapping test of outer loadings (Mean, STDEV, T-values)

Manifest variable	Original Sample (O)	Sample Mean (M)	Standard Deviation (STDEV)	Standard Error (STERR)	T Statistics (|O/STERR|)
x1 <- TB Epidemic	0.6405	0.6152	0.3008	0.3008	2.1296**
x2 <- TB Epidemic	0.8219	0.7174	0.3057	0.3057	2.6888**
x3 <- TB Epidemic	0.8219	0.7161	0.3062	0.3062	2.6846**
x4 <- Health service	0.8626	0.2554	0.6019	0.6019	1.4331*
x5 <- Health service	0.5313	0.5564	0.4203	0.4203	1.2641
x6 <- Health service	0.5313	0.5545	0.4228	0.4228	1.2566
x7 <- Health service	0.5772	0.5699	0.4273	0.4273	1.3509*
x8 <- Health Expenditure	0.8209	0.6921	0.3694	0.3694	2.2220**
x9 <- Health Expenditure	0.9977	0.8866	0.3280	0.3280	3.0418**
x10 <- Health Expenditure	0.9857	0.8734	0.3400	0.3400	2.8995**
x11 <- DOTS Effect	0.8871	0.8834	0.0545	0.0545	16.2772**
x12 <- DOTS Effect	0.9057	0.9033	0.0467	0.0467	19.4104**
AP <- Humidity	0.8636	0.8586	0.0421	0.0421	20.4883**
GCZ <- Humidity	0.9375	0.9378	0.0137	0.0137	68.4549**
ATT <- Temperature	0.9679	0.9653	0.0626	0.0626	15.4580**
GL <- Tem Temperature	0.9424	0.9377	0.0643	0.0643	14.6578**
TCZ <- Tem Temperature	0.9457	0.9436	0.0618	0.0618	15.2957**
MDR-rate <- DR-TB	0.8960	0.8905	0.0236	0.0236	38.0375**
Mono-rate <- DR-TB	0.6749	0.6855	0.0618	0.0618	10.9152**
Poly-rate <- DR-TB	0.9134	0.9129	0.0205	0.0205	44.4736**

**P < 0.05, *P < 0.20.

**Table 4:** Bootstrapping test of path coefficients (Mean, STDEV, T-values)

Latent variable	Original Sample (O)	Sample Mean (M)	Standard Deviation (STDEV)	Standard Error (STERR)	T Statistics (|O/STERR|)
TB Epidemic -> DR-TB	0.1858	0.1455	0.1272	0.1272	1.4606*
Health service -> DR-TB	0.0875	0.0316	0.1918	0.1918	0.4563
Health Expenditure -> DR-TB	-0.2791	-0.2770	0.2085	0.2085	1.3385*
DOTS Effect -> DR-TB	0.1646	0.1500	0.0741	0.0741	2.2201**
Humidity -> DR-TB	-0.3515	-0.3610	0.0830	0.0830	4.2347**
Temperature -> DR-TB	-0.3358	-0.3477	0.1120	0.1120	2.9990**

**P < 0.05, *P < 0.20.

### Global spatial dependence between "DR-TB" and latent synthetic risk factors

The result of OLS regression between "DR-TB" and latent synthetic risk factors (Table 5) showed that "DR-TB" was significantly associated with latent synthetic risk factors (*F*=*19.28, P *< 0.0001), and explained about 33.50% of the total variance of "DR-TB" (adjusted *R*^2 ^= 0.3350). Moreover, the hypothesis test of the partial regression coefficient (Table 5) demonstrated that the higher "TB Epidemic" and "DOTS Effect", and lower "Health Expenditure", "Humidity" and "Temperature", corresponded with higher "DR-TB"; but the relationship between "Health Service" and "DR-TB" was not statistically significant (*P *= 0.7991). It can be seen that the OLS regression result was similar to that of the PLS path modeling, which, to some extent, demonstrated the fitness and accuracy of Kriging interpolation.

**Table 5:** Parameter estimates of the OLS regression model

Variable	DF	Parameter Estimate	Standard Error	t Value	Pr > |t|
Intercept	1	-0.04735	0.04701	-1.01	0.3150
TB Epidemic	1	0.13681	0.08082	1.69	0.0920
Health Service	1	0.01824	0.07159	0.25	0.7991
Health Expenditure	1	-0.18724	0.07877	-2.38	0.0184
DOTS Effect	1	0.19963	0.06700	2.98	0.0032
Humidity	1	-0.36607	0.05559	-6.58	<.0001
Temperature	1	-0.21369	0.05542	-3.86	0.0002

*R*^2 ^= 0.353, adjusted *R*^2 ^= 0.335, *AIC*c = 470.952.

### Local spatial dependence between "DR-TB" and latent synthetic risk factors

Table 6 summarizes the results of GWR between "DR-TB" and latent synthetic risk factors, and indicated that there was large spatial variability in the parameter estimates from different regions' models. An increase in the adjusted *R*^2 ^was found, i.e., from 0.335 (OLS) to 0.592 (GWR), which demonstrated that the GWR model had a much better explanatory power than the OLS model. In addition, the result of the *F*-test (*F *= 7.0899, *P *< 0.05) also suggested that the improvement in model fit using GWR was statistically significant. Furthermore, based on the evaluation criterion of the GWR model suggested by Fotheringham [20], the best model was the one with the smallest *AIC*c value; and as a rule of thumb, a "serious" difference between two models is generally regarded as one where the difference in *AIC*c values between the models is at least 3. In this study, the *AIC*c of GWR (394.851) was far smaller than in the OLS (470.952), which illustrated that the GWR model was better than the OLS model.

**Table 6:** Parameter estimates of the GWR model

Parameter	Minimum	1st Quartile	Median	3rd Quartile	Maximum
Constant	-0.41328	-0.19868	-0.05409	0.14986	0.54743
TB Epidemic	-0.08524	0.02247	0.13649	0.26689	0.47369
Health service	-0.2734	0.00381	0.05076	0.15266	0.28677
Health Expenditure	-0.87021	-0.52551	-0.28754	-0.11999	0.05633
DOTS Effect	-1.14011	-0.01533	0.09909	0.14826	0.25874
Humidity	-0.88509	-0.51084	-0.3335	-0.2001	-0.00262
Temperature	-0.88254	-0.42387	-0.28993	-0.13151	0.22495

*R*^2 ^= 0.641, adjusted *R*^2 ^= 0.592, *AIC*c = 394.851.

**Figure 3:**
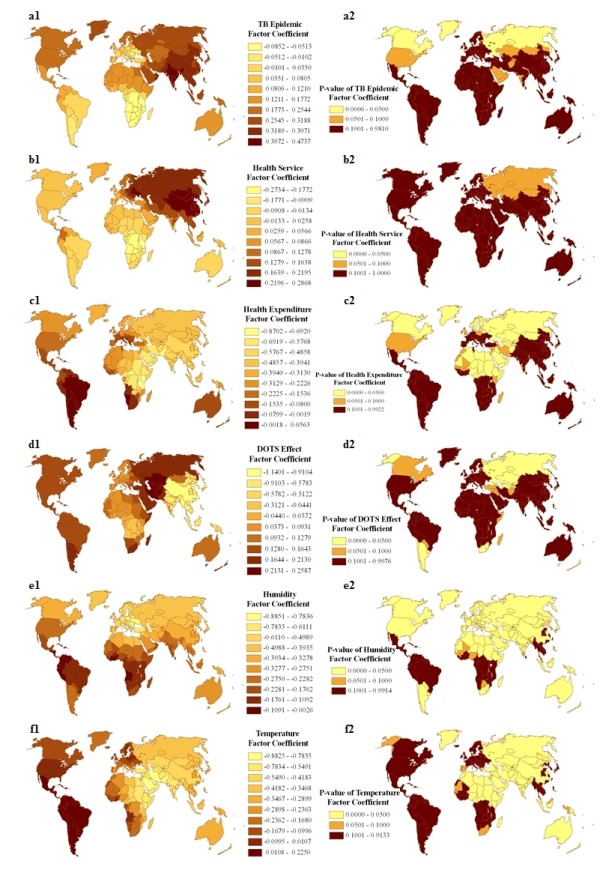
**Worldwide geographic clines of six latent synthetic risk factor coefficients**. a1-f1: Distribution of "TB Epidemic", "Health Service", "Health Expenditure", "DOTS Effect", "Humidity" and "Temperature" factor coefficients, respectively, derived from the GWR model. a2-f2: *P*-value distribution of six synthetic latent factor coefficients derived from the GWR model.

Figure 3 shows the contour map of the regression coefficients of six latent synthetic risk factors and their *P *values, interpolated by the Kriging method. It is clear that the regression coefficients varied spatially, and the local spatial dependence relationship between "DR-TB" and the six latent synthetic risk factors exhibited a non-constant mean and variance across the whole world. This suggests a non-stationary relationship between "DR-TB" and the latent synthetic risk factors. The standardized regression coefficient estimates of "TB Epidemic" were mostly positive (some were not statistically significant), except in southern South America, eastern and southern Europe, and central and southern Africa (see Figure 3a1, a2). In contrast, there was a negative association in southern South America (Chile, Argentina, Paraguay and Uruguay), eastern and southern Europe (Ukraine, Romania, Bulgaria, Slovakia, Czech Republic, Austria, Hungary, Switzerland, Italy, Greece, etc), and central and southern Africa (Gabon, Congo, Uganda, Kenya, Tanzania, Angola, Zambia, Malawi, Mozambique, Zimbabwe, Namibia, Botswana, Swaziland, Lesotho, South Africa, etc), but none of these was statistically significant. The standardized regression coefficient estimates of "Health Service" were mostly positive except in the USA, Mexico, eastern and southern South America, central and southern Africa, Australia, and New Zealand (see Figure 3b1, b2). However, only the regression coefficients of Russia, Kazakhstan and Mongolia were statistically significant. In eastern and southern South America (Brazil, Bolivia, Chile, Argentina, Paraguay, Uruguay) and Namibia, the standardized regression coefficient estimates of "Health Expenditure" were positive, and the rest were negative, indicating a negative association between "DR-TB" and "Health Expenditure" in most regions of the world, but some were not statistically significant (see Figure 3c1, c2). In China, Japan, India, Southeast Asia and central Africa, the standardized regression coefficients estimates of the "DOTS Effect" were negative, and the rest were positive, but some were not statistically significant (see Figure 3d1, d2). The standardized regression coefficient estimates of "Humidity" were negative in all regions, but some were not statistically significant (see Figure 3e1, e2). A negative association between "DR-TB" and "Temperature" was revealed in most areas of the world (some were not statistically significant) except in Latin America (see Figure 3f1, f2).

## Discussion

As a serious public health and social problem worldwide, DR-TB and its effective prevention and control has become a hot issue. While there are wide areas of development in the treatment of DR-TB (drug therapy, immunotherapy, gene therapy, etc), it is not an easy problem to overcome. Therefore, prevention and control measures should be taken for DR-TB. It is crucial to explore the risk factors of DR-TB, especially the ecological factors. Nonetheless, at present, there has been no specialized research published to explore the ecological factors in DR-TB. In this study, we conducted such a study using anti-tuberculosis drug resistance data from the WHO/UNION Global Project and the collected ecological factors data. An ecological study often lacks the ability to control potential confounding factors, and ecological fallacy is inevitable. Therefore, we selected all the anti-tuberculosis drug resistance countries or regions as study samples, and implemented strict quality control measures (including risk factor variable determination and collection, data sorting and analysis, etc.) to improve the research quality as far as possible.

Previous studies, such as researches that explored the ecological factors of TB [4,5,39-44], were all based on data distribution to construct a linear regression model, a Poisson regression model or a logistic regression model directly to estimate the relationship between factors and TB incidence rate, without considering the internal relevance and potential structure of the factors. With the unit of anti-tuberculosis drug resistance monitoring countries or regions, we explored the relationship between ecological factors (TB epidemic, DOTS effect, health expenditure, climatic geography, etc.) and the level of DR-TB globally. In order to fully use the data information and reveal the inner characteristics of risk factors comprehensively and thoroughly, EFA was used to find the latent synthetic risk factors, and then PLS path modeling was constructed to show the complex ecologic causal relationship between the level of DR-TB and the latent synthetic risk factors. Finally, six latent factors ("TB Epidemic", "Health Service", "Health Expenditure", "DOTS Effect", "Humidity" and "Temperature") were extracted, and "DR-TB" was used to reflect the level of the DR-TB rate. We found that except the "Health Service" factor, other factors had a major impact on "DR-TB".

According to the predicated values of "DR-TB" and the latent synthetic risk factors by the Kriging method, the GWR model was constructed to analyze the local relationship between latent synthetic risk factors and "DR-TB". The parameters of the GWR model in different regions reflected the influence of the degree and direction of association of each latent synthetic factor to "DR-TB". The contour map of the regression coefficients in the GWR model showed that it had significant spatial variability, which demonstrated that the effect of latent synthetic risk factors on the level of DR-TB was different in different regions. The results suggested that local control and prevention strategies and monitoring schemes should be formulated according to the spatial characteristics of the latent synthetic risk factors and the local association with drug-resistance rates, instead of roughly establishing global strategies and policies based only on the results of drug-resistance monitoring.

The GWR model is an expansion of traditional regression analysis, which allows the parameters to vary with changes in spatial location. Unlike traditional methods, the GWR model can adjust the spatial heterogeneity by changing the sample location of spatial data, and then estimate the local parameter to reflect the variance of the factor contribution in different areas, hence its regression results are much more reasonable [45]. In the present study, the relationships between "DR-TB" and latent synthetic risk factors showed spatial variability, thus the GWR model was chosen for local estimation. The GWR results showed that the signs of parameter estimates are not all the same as those in the OLS regression model. Also the average adjusted *R*^2 ^(0.592) of the GWR model was larger than the *R*^2 ^(0.335) of the OLS model, which reflected the spatial variability of DR-TB. Meanwhile, the *AIC*c (394.851) of the GWR model was smaller than that of the OLS model (470.952), which also demonstrated that the fit of the GWR model was better than that of the OLS model.

## Conclusions

This study found that GWR (local) showed a stronger relationship between latent synthetic risk factors and the DR-TB latent factor than OLS (global) regression, and established the "non-stationary" nature of the local relationship, i.e., the influence of the latent synthetic risk factors on DR-TB presented spatial variability. Thus, monitoring planning and prevention and control strategies for DR-TB should be formulated according to the spatial characteristics of the latent synthetic risk factors and the local relationship between "DR-TB" and the latent synthetic risk factors.

## List of abbreviations used

*AIC*c: corrected Akaike's Information Criterion; AP: annual precipitation; ATT: annual atmospheric temperature; DOTS: directly-observed treatment strategy; DR-TB: drug-resistant tuberculosis; DTP3: diphtheria-tetanus-pertussis; E: ethambutol; EFA: exploratory factor analysis; GCZ: geography climatic zone; GL: geography latitude; GWR: geographical weighted regression; H: isoniazid; MCV: meningococcal conjugate vaccine; MDR-TB: multidrug-resistant tuberculosis; OLS: ordinary least square; SEM: structural equation model; PLS-PM: partial least square path modeling; R: rifampicin; S: streptomycin; TB: tuberculosis; TCZ: temperature climate zone; WHO/UNION: World Health Organization/International Union Against Tuberculosis and Lung Disease.

## Competing interests

The authors declare that they have no competing interests.

## Authors' contributions

YXL extracted the data, conducted the statistical analysis and drafted the manuscript. SWJ helped with the study design and interpretation of the results. YL helped to interpret the results and modify the manuscript. RW extracted the data. XL extracted the data and assisted with the statistical analysis. ZSY helped write and modify the manuscript. LXW helped to interpret the results. FZX conceived of the project concept, assisted with the data interpretation, and helped write the manuscript. All of the authors have read and approved the final manuscript.
